# Development of Cu@Zr-MOFs-PAN Nanofiber Composites for Efficient Methylene Blue Adsorption in Wastewater Treatment

**DOI:** 10.3390/polym17172404

**Published:** 2025-09-03

**Authors:** Zibin Li, Lizhen Zhang, Guoyuan Yuan

**Affiliations:** 1 School of Emergency Management, Chongqing Vocational Institute of Safety Technology, Chongqing 401331, China; keyangongzuo521@163.com; 2 College of Chemistry and Chemical Engineering, Chongqing University of Science and Technology, Chongqing 401331, China

**Keywords:** MOFs, nanofiber, methylene blue, removal

## Abstract

Methylene blue (MB) is a commonly used dye that generates a large amount of dye wastewater during its application. If discharged untreated, it poses a serious threat to water environments and human health. Therefore, the removal of methylene blue from dye wastewater is crucial. In this study, we used zirconium-based metal–organic frameworks (Zr-MOFs) as a precursor and doped them with copper ions to prepare the Cu@Zr-MOFs composite material. Subsequently, we fabricated Cu@Zr-MOFs-PAN nanofiber composites through electrospinning to address the challenge of separating Cu@Zr-MOFs from water. The results indicate that the introduction of copper ions significantly enhances the adsorption capacity of Zr-MOFs for MB, increasing the adsorption amount from 158.0 mg/g to 266.0 mg/g, representing a 68.3% improvement. Furthermore, the prepared Cu@Zr-MOFs-PAN nanofibers exhibited an MB adsorption capacity of 162.1 mg/g, further confirming the successful preparation of Cu@Zr-MOFs-PAN. X-ray photoelectron spectroscopy (XPS) analysis shows that copper doping not only enhances the structural stability of the material but also increases the density of active sites for MB adsorption. This study not only provides an efficient material for the removal of MB from wastewater but also offers an important theoretical basis for the design of novel nanomaterials for environmental pollutant remediation.

## 1. Introduction

Methylene blue (MB) is a common azo dye extensively employed across diverse sectors, including textiles and printing [1,2,3]. However, the production and use of MB often generate significant amounts of wastewater. This wastewater typically exhibits a certain degree of biotoxicity, and if discharged untreated, it can inflict substantial harm on aquatic ecosystems and present risks to human health [4,5]. Consequently, the separation of MB from wastewater is of paramount significance.

The primary methods for separating MB include adsorption and catalytic degradation [6,7,8]. Among these, adsorption is favored due to its efficiency, environmental friendliness, and technical flexibility, allowing for deep purification and rapid separation of MB. As a result, adsorption is widely employed in research aimed at MB separation. Common adsorbent materials include graphene [4] and polymeric materials [3]. However, these materials often face challenges, such as low adsorption capacity and difficulties in quickly separating the adsorbent from the solution. Therefore, the development of novel adsorbent materials is critical for enhancing MB separation.

In recent years, nanofibers produced through electrospinning technology have garnered significant attention in the field of wastewater treatment. This interest arises from the fact that, compared to traditional powder adsorbents, nanofibers possess a larger specific surface area and a favorable pore structure, which facilitates their rapid separation from aqueous solutions during use [9,10]. However, pure nanofibers typically demonstrate lower adsorption capacities due to a limited number of active sites on their surfaces. To address this limitation, researchers often incorporate fillers with good adsorption properties into the nanofibers, such as carbon dots [11], graphene [12], carbon nanotubes [13], and metal oxides [14], which can significantly enhance the adsorption performance of the fibers. Additionally, chemical modifications can introduce functional groups onto the surface or within the fibers, providing extra adsorption sites and further improving their adsorption capabilities [15]. For instance, Lee et al. [16] utilized electrospinning technology to prepare polycaprolactone/polyvinylpyrrolidone (PCL/PVP) composite fibers, where the ratio of PCL to PVP was finely controlled to adjust the fiber morphology and specific surface area. After doping with ZIF-8, the fibers achieved an adsorption amount of up to 151.6 mg/g for methylene blue. Similarly, a dual-functional nanofiber composite membrane developed by Li et al. exhibited 152.4 mg/g for urea and 100.5 mg/g for creatinine [17]. Thus, modifying nanofibers is crucial for enhancing their adsorption performance for MB.

Metal–organic frameworks (MOFs) constitute an innovative category of porous materials with substantial applications in gas storage [18,19], catalysis [20,21,22], sensing [23,24] and separation [25,26]. MOFs feature extensive specific surface areas and customizable pore architectures, and they can be chemically modified to introduce specific functional groups, significantly enhancing their adsorption capacity for metal ions and organic pollutants [27,28]. However, MOFs still face challenges in practical applications, such as particle aggregation, difficulties in separation and recovery from solutions, and potential structural degradation in complex wastewater environments. Encapsulating MOFs in nanofibers can effectively address these issues. Furthermore, pristine MOFs exhibit relatively low adsorption capacity for MB, while the incorporation of copper ions can significantly enhance their adsorption performance, which is primarily attributed to multiple synergistic mechanisms [2,29,30]. Firstly, the substitution of Cu^2+^ ions for the original metal nodes in MOFs can modulate the surface charge of the material, thereby strengthening the electrostatic adsorption of positively charged MB molecules. Secondly, copper ions synergize with functional groups in MOFs to improve adsorption performance through hydrogen bonding and π-π interactions. Additionally, copper doping can generate more coordinatively unsaturated metal sites, which chemically coordinate with the sulfonate groups or nitrogen atoms in MB.

Herein, this study utilizes zirconium-based MOFs as precursors to construct Cu@Zr-MOFs composite materials through copper ion doping. The doped copper ions are expected to increase the number of active adsorption sites within the Zr-MOFs and optimize their electronic structure, thereby significantly enhancing the adsorption performance of the MOFs for MB. Subsequently, Cu@Zr-MOFs will be employed as fillers, with polyacrylonitrile (PAN) serving as the matrix, to prepare copper-doped MOF nanofibers (Cu@Zr-MOFs-PAN) using electrospinning technology. This composite material will also be utilized in the separation of methylene blue, providing a novel approach for the effective removal of MB from wastewater.

## 2. Experimental

### 2.1. Chemicals

The main reagents required for the experiments, including MB, terephthalic acid (TPA), ZrCl_4_, Cu(NO_3_)_3_·3H_2_O, and polyacrylonitrile (PAN), polyvinyl pyrrolidone (PVP), were obtained from Macklin Chemical Co., Ltd. (Shanghai, China).

### 2.2. Fabrication of Cu@Zr-MOFs-PAN

Dissolve 0.6 g of Cu(NO_3_)_2_, 2.33 g of ZrCl_4_, and 1.66 g of TPA in 100 mL of DMF. Stir the mixture thoroughly, then transfer it to a reactor and maintain a temperature of 120 °C for 48 h. After the reaction is complete, allow the mixture to cool to room temperature, then wash and dry the resulting product to obtain Cu@Zr-MOFs. The preparation of Zr-MOFs follows the same procedure as that for Cu@Zr-MOFs, but without the addition of Cu(NO_3_)_2_.

Add 0.4 g of Cu@Zr-MOFs, 0.2 g of PVP, and 0.4 g of PAN in 5 mL of DMF, stirring thoroughly to ensure a homogenous solution. Next, process this solution using an electrospinning device to create fibers. After the electrospinning process, wash the fibers with deionized water to remove PVP, resulting in the formation of Cu@Zr-MOFs-PAN nanofibers. Detailed information about the electrospinning parameters can be found in Section 1 of the Supporting Information (SI).

### 2.3. Characterization Equipment

The characterization equipment used is detailed in Section 2 of the Supporting Information.

### 2.4. Adsorption Experiment

In total, 20 mg of the adsorbent was added into a flask containing 100 mL of an MB solution. Adjust the pH using either NaOH or HNO_3_ (0.1 mol/L). Subject the mixture to agitation on a thermostatically controlled shaker at 150 rpm until the predetermined time is reached. After the adsorption equilibrium is achieved, filter a measured volume of the solution through a hydrophilic filter membrane, and then determine the MB content using a spectrophotometer. To ensure the reliability of the experimental results, each experimental condition is replicated three times. Calculate the average adsorption capacity *q*_e_ (mg/g) using Equation (1) [31], where *C*_0_ and *C*_e_ represent the initial and equilibrium concentrations of MB (mg/L), respectively, *V* denotes the solution volume (L), and *m* represents the mass of the adsorbent (g).

(1) $$q_{e}=\frac{(C_{0}-C_{e})\times V}{m}$$

Dynamic adsorption experiments were conducted using a chromatography column (diameter 15.6 mm) packed with 2.0 g of Cu@Zr-MOFs-PAN. The column was first flushed with deionized water at pH 7 to remove any trapped air in the tubing. Subsequently, an MB solution with a concentration of 100 mg/L and pH 7 was introduced into the column at a flow rate of 0.5 mL/min using a peristaltic pump. The effluent was collected and analyzed for MB concentration with a spectrophotometer. The dynamic adsorption capacity *q*_f_ (mg/g) for Cu@Zr-MOFs-PAN was calculated according to Equations (S1) and (S2) [32].

## 3. Results and Discussion

### 3.1. Characterization

**Figure 1:**
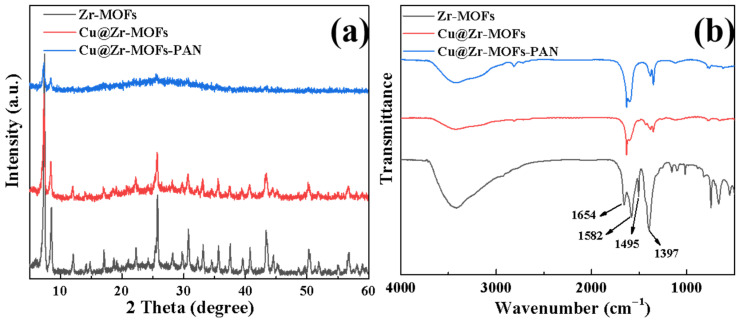
XRD (**a**) and FT-IR (**b**) characterization of Zr-MOFs, Cu@Zr-MOFs, and Cu@Zr-MOFs-PAN.

Figure 1a displays the XRD patterns of Zr-MOFs, Cu@Zr-MOFs, and Cu@Zr-MOFs-PAN composite materials. The XRD pattern of Zr-MOFs displays distinctive diffraction peaks that correspond precisely with those of UiO-66, thereby confirming the successful synthesis of Zr-MOFs, as demonstrated by their remarkable concordance with existing literature [32,33]. Upon doping with copper ions to form Cu@Zr-MOFs, significant changes are observed in the XRD characteristics compared to pristine Zr-MOFs: the intensities of characteristic diffraction peaks are generally reduced, and some characteristic peaks (e.g., at 2θ = 14°) completely disappear. This phenomenon indicates that the introduction of copper ions leads to partial coverage of the Zr-MOFs by copper species, which predominantly exist in an amorphous state within the framework.

When Cu@Zr-MOFs are further incorporated into PAN nanofibers to form the composite, the XRD diffraction signals exhibit a pronounced intensity reduction, primarily due to two factors: (1) the relatively low mass fraction of Cu@Zr-MOFs in the composite system (8 wt%); and (2) the partial encapsulation effect of the PAN matrix, which attenuates the diffraction signals from the Cu@Zr-MOFs particles. Notably, despite the reduced signal intensity, characteristic diffraction peaks originating from Cu@Zr-MOFs remain observable, providing direct evidence for the structural composition of the composite material. Based on the comprehensive analysis above, this study successfully prepared Cu@Zr-MOFs-PAN composite nanofibers.

Figure 1b illustrates the FT-IR characterization results of Zr-MOFs, Cu@Zr-MOFs, and Cu@Zr-MOFs-PAN composite materials. The absorption peak at 1654 cm^−1^ corresponds to the stretching vibration of C=O, the peak at 1582 cm^−1^ is attributed to the asymmetric stretching vibration of the carboxyl group on the benzene ring, the peak at 1495 cm^−1^ reflects the stretching vibration of the C=C bond in the aromatic ring, and the peak at 1397 cm^−1^ corresponds to the symmetric stretching vibration of the O-C-O bond in the BDC ligand [1,25]. The positions and intensities of these characteristic peaks are highly consistent with the standard spectrum of UiO-66 reported in the literature, which fully confirms the successful synthesis of Zr-MOFs.

Upon doping with copper ions, the FT-IR spectrum of Cu@Zr-MOFs exhibits significant changes: the intensities of most characteristic peaks generally decrease, and some characteristic peaks undergo a blue shift. This indicates that the introduction of copper ions causes partial disruption of the Zr-MOFs crystal structure, with copper species likely existing in an amorphous or coordinated state within the framework.

After further loading Cu@Zr-MOFs onto PAN nanofibers, the FT-IR spectrum of the resulting Cu@Zr-MOFs-PAN composite material shows that the intensities of the original characteristic peaks are further weakened. This is mainly due to the low mass fraction of Cu@Zr-MOFs in the composite system and the partial encapsulation effect of the PAN matrix on the Cu@Zr-MOFs particles, leading to attenuation of the infrared absorption signal. Nonetheless, the characteristic peaks of Cu@Zr-MOFs remain clearly discernible, providing direct evidence for the structural composition of the composite material. Based on the aforementioned analysis, this study successfully prepared Cu@Zr-MOFs-PAN composite nanofibers with a core–shell structure.

**Figure 2:**
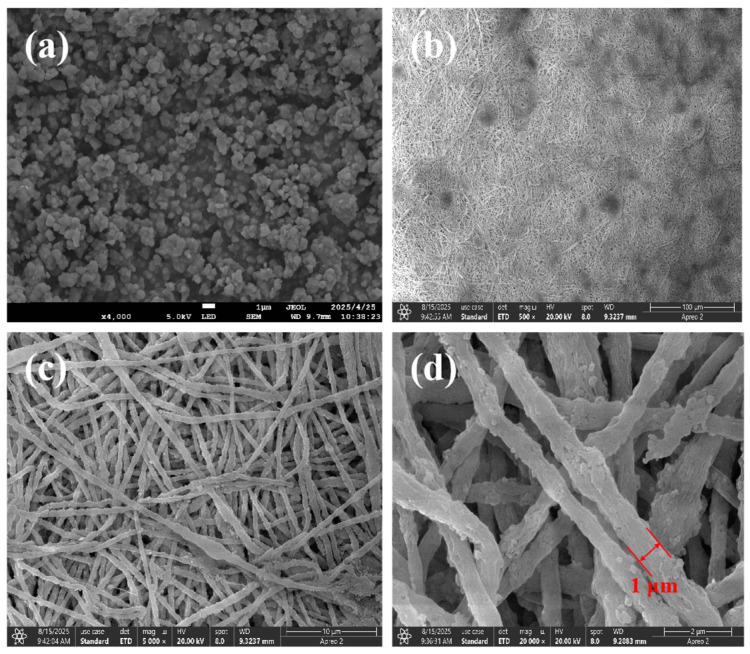
SEM characterization of Cu@Zr-MOFs (**a**), and Cu@Zr-MOFs-PAN (**b**–**d**).

Figure 2 presents the SEM characterization of Cu@Zr-MOFs (a) and Cu@Zr-MOFs-PAN composite nanofibers (b–d). As illustrated in Figure 2a, Cu@Zr-MOFs exhibit a well-defined crystalline structure with a spherical morphology. Upon fabrication into nanofibers, it is evident that the Cu@Zr-MOFs are encapsulated within the PAN fibers, forming a composite architecture. The diameter of the fibers is approximately 1 μm. This observation suggests successful integration of the MOF nanoparticles within the polymer matrix, which may impart enhanced functionalities to the resulting composite material.

Figure S1 displays the nitrogen adsorption–desorption isotherms of Zr-MOFs, Cu@Zr-MOFs, and Cu@Zr-MOFs-PAN. Table 1 summarizes the pore structure data for the three materials. As indicated in the table, Zr-MOFs exhibit a specific surface area (SSA) of 832.1 m^2^/g, while Cu@Zr-MOFs show a reduced value of 435.0 m^2^/g. This decrease in surface area supports the presence of copper in an amorphous state within the material. Upon fabrication into nanofibers, the specific surface area of Cu@Zr-MOFs-PAN further decreases to 80.2 m^2^/g. Additionally, the pore volume (PV) of Zr-MOFs is 0.32 cm^3^/g, which further diminishes in Cu@Zr-MOFs, providing further evidence of the amorphous nature of copper species within the framework.

### 3.2. Adsorption Properties

#### 3.2.1. pH

**Figure 3:**
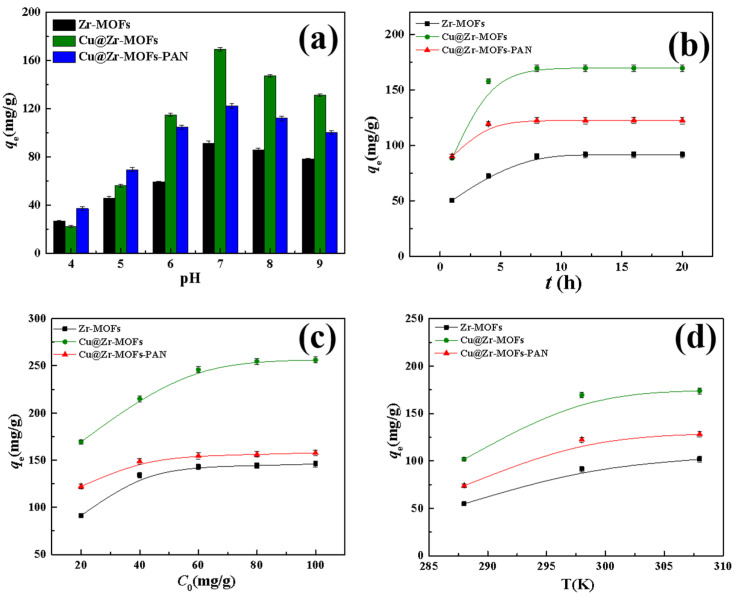
Influence of various parameters on the adsorption efficacy of Zr-MOFs, Cu@Zr-MOFs, and Cu@Zr-MOFs-PAN for MB: (**a**) pH, (**b**) contact duration, (**c**) initial concentration, and (**d**) temperature.

Figure 3a shows the adsorption performance of Zr-MOFs, Cu@Zr-MOFs, and Cu@Zr-MOFs-PAN on MB at different pH values. The data indicate that all three materials reach their maximum adsorption capacity at pH 7. Cu@Zr-MOFs exhibit an adsorption capacity of 169.4 mg/g, significantly higher than that of undoped Zr-MOFs (91.5 mg/g). This improvement is attributed to Cu doping, which enhances the electronic structure of Zr-MOFs, increases surface charge density, and provides more active sites [34,35]. The strong interaction between Cu’s d-electrons and the π-electrons of MB molecules strengthens the adsorption capacity and optimizes the material’s surface acidity and basicity [36], making it more effective at pH 7.

Although the adsorption capacity of Cu@Zr-MOFs-PAN decreases to 122.4 mg/g, this is not a decline in performance. With only 8% Cu@Zr-MOFs content, the composite shows efficient utilization of Cu@Zr-MOFs in practical applications. The incorporation of Cu@Zr-MOFs into PAN substantially improves the overall adsorption efficacy of the composite, emphasizing the importance of Cu@Zr-MOFs in the matrix. Given that pH 7 is optimal for adsorption, the pH level established was maintained for all subsequent experiments.

#### 3.2.2. Contact Time

Figure 3b depicts the time-dependent adsorption behaviors of Zr-MOFs, Cu@Zr-MOFs, and Cu@Zr-MOFs-PAN. Zr-MOFs reach equilibrium after 12 h with a relatively slow adsorption rate, which is likely due to their simple pore structure that limits the interaction opportunities between MB molecules and adsorption sites, and they may also have a limited number of active surface sites. In contrast, Cu@Zr-MOFs adsorb more than 90% of MB within 4 h and reach equilibrium after 8 h. The enhanced adsorption rate is attributed to Cu doping, which alters the electronic structure of Zr-MOFs, enabling strong p-π conjugation or electrostatic interactions with MB, and Cu also introduces new active sites to improve adsorption efficiency [35]. After embedding Cu@Zr-MOFs into PAN, the adsorption rate of Cu@Zr-MOFs-PAN increases significantly compared to pristine Cu@Zr-MOFs, mainly because the unique porous fibrous structure of PAN facilitates the rapid diffusion of MB molecules into the adsorbent matrix. Although the PAN matrix requires MB molecules to traverse an additional diffusion path before reaching the Cu@Zr-MOFs surface, this minor diffusion resistance is outweighed by the enhanced mass transport properties of the composite structure. As a result, Cu@Zr-MOFs-PAN achieves over 95% adsorption capacity within 4 h and reaches equilibrium within 8 h, demonstrating its efficient adsorption performance. The PAN matrix, while increasing resistance to diffusion, offers a longer residence time for MB molecules, enhancing adsorption.

**Figure 4:**
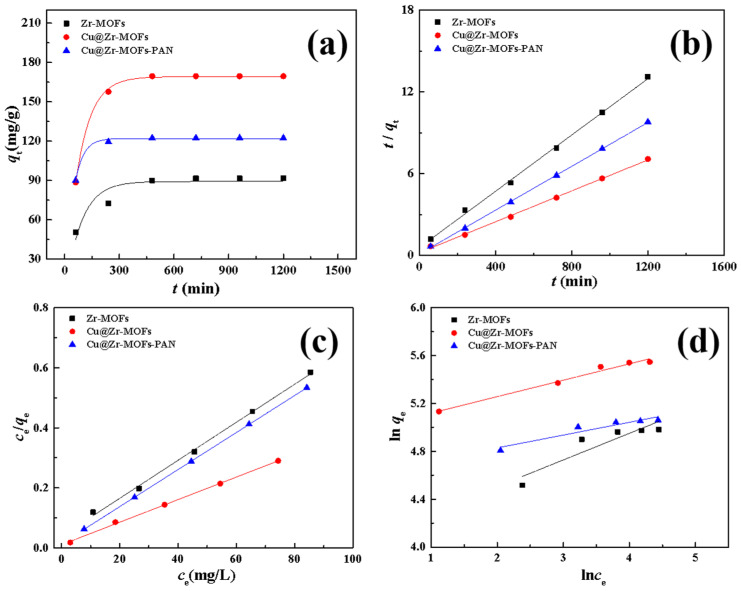
Fitted kinetic models for (**a**) PFO, (**b**) PSO; isotherm models for (**c**) Langmuir, and (**d**) Freundlich.

To further explore the adsorption mechanism of the materials for MB, pseudo-first-order (PFO) and pseudo-second-order (PSO) kinetic models [32] were fitted (Figure 4a,b) to the experimental data using Equations (S3) and (S4) in the SI. The kinetic analysis (Table 2) reveals distinct adsorption behaviors among the three materials. Zr-MOFs exhibit a superior fit to the PSO model, confirming that chemisorption constitutes the predominant mechanism. Notably, both Cu@Zr-MOFs and Cu@Zr-MOFs-PAN demonstrate comparable high correlation coefficients for both PFO and PSO models, suggesting a synergistic interplay between physical and chemical adsorption processes [37]. The enhanced chemisorption capacity originates from the strategic doping of Cu and the hierarchical composite architecture. The embedded Cu atoms serve as active sites that form coordinate bonds with the nitrogen-containing functional groups of MB, while the porous fibrous structure of the composite facilitates initial physical adsorption through π-π stacking interactions between the aromatic rings of MB and the MOF framework [34]. This dual-action mechanism not only accelerates the initial adsorption rate but also strengthens the overall binding affinity, as reflected by the rapid 95% removal efficiency achieved within 4 h.

#### 3.2.3. Initial MB Concentration

Figure 3c shows the adsorption of MB by Zr-MOFs, Cu@Zr-MOFs, and Cu@Zr-MOFs-PAN at various initial concentrations. The adsorption amount increases with the initial MB concentration until saturation, at which point the adsorption stabilizes. At lower concentrations, the number of MB molecules is limited, and the adsorption is controlled by diffusion from the solution to the adsorbent surface. As the concentration increases, the diffusion force strengthens, allowing MB molecules to reach the adsorption sites more effectively. However, once all active sites are occupied, further concentration increases do not significantly impact adsorption.

To understand the adsorption behavior and mechanisms, the experimental data were fitted (Figure 4c,d) to the Langmuir and the Freundlich isothermal models [37,38] using Equations (S5) and (S6) in the SI. As shown in Table 3, the adsorption behavior of all three materials closely follows the Langmuir model, as indicated by higher R^2^ values. This suggests a monolayer, homogeneous adsorption mechanism. Based on Langmuir model calculations, the maximum adsorption capacities for MB are 158.0 mg/g for Zr-MOFs, 266.0 mg/g for Cu@Zr-MOFs, and 162.1 mg/g for Cu@Zr-MOFs-PAN. The significant increase in Cu@Zr-MOFs’ adsorption capacity compared to Zr-MOFs is due to Cu doping, which provides additional active sites and enhances surface charge density. The relatively high adsorption capacity of Cu@Zr-MOFs-PAN further validates the composite’s performance, combining Cu@Zr-MOFs’ high adsorption with the structural benefits of PAN nanofibers. Moreover, the obtained Cu@Zr-MOFs-PAN exhibits a relatively high adsorption capacity for methylene blue compared to various other methylene blue adsorbent materials (Table 4).

#### 3.2.4. Temperature

Figure 3d examines the adsorption of MB by Zr-MOFs, Cu@Zr-MOFs, and Cu@Zr-MOFs-PAN at varying temperatures. The uptake amount increases with temperature, indicating that higher temperatures facilitate the adsorption process. At low temperatures, weak molecular thermal motion results in slower diffusion and limited interactions between MB molecules and the adsorbent surface. Higher temperatures enhance molecular motion, promote faster diffusion, and reduce energy barriers, allowing more MB molecules to adsorb onto the material.

To gain deeper insight into the thermodynamic properties of the adsorption process, this study applied classical thermodynamic equations (Equations (S7) and (S8)) to calculate key thermodynamic parameters [39,40], as shown in Table 5. The data clearly demonstrate that the ΔG^0^ values for all three materials are negative during the adsorption of MB. According to thermodynamic principles, a negativeΔG^0^ indicates a spontaneous process, meaning that the adsorption of MB by Zr-MOFs, Cu@Zr-MOFs, and Cu@Zr-MOFs-PAN occurs spontaneously within the studied temperature range, without the need for external energy input. Additionally, positive ΔH^0^ values suggest that the adsorption process is endothermic, with heat absorption strengthening the interactions between MB and the adsorbent surface. As the temperature increases, the thermal energy enhances the adsorption capacity.

### 3.3. Dynamic Adsorption Study

**Figure 5:**
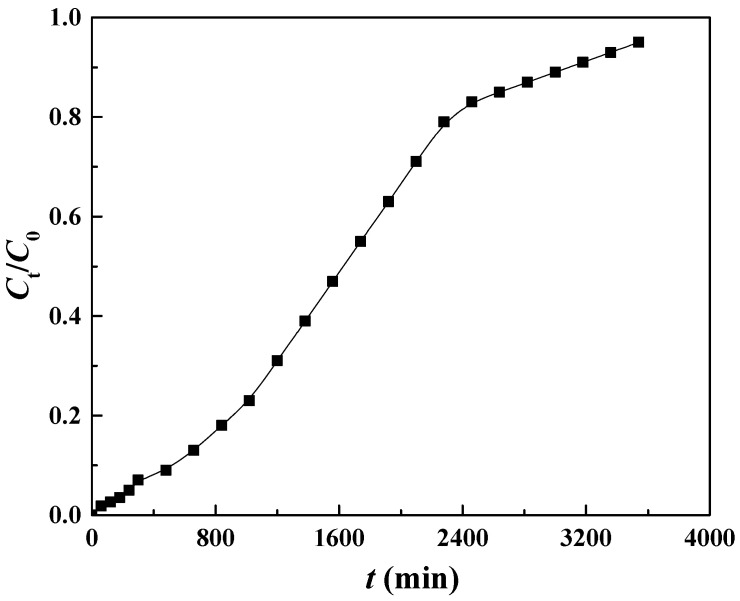
Research on fixed-bed adsorption of MB by Cu@Zr-MOFs-PAN.

The dynamic adsorption experiment was conducted in a fixed-bed system to assess the adsorption capacity of Cu@Zr-MOFs-PAN for MB. The breakthrough curve (Figure 5) revealed that during the first 240 min, the MB concentration in the effluent remained below 0.05 times the initial concentration, indicating excellent adsorption efficiency. After 240 min, the effluent concentration exceeded this threshold, marking the breakthrough point. From 240 to 3540 min, the concentration gradually increased, indicating reduced adsorption capacity, and by 3540 min, the adsorbent’s capacity was exhausted.

**Figure 6:**
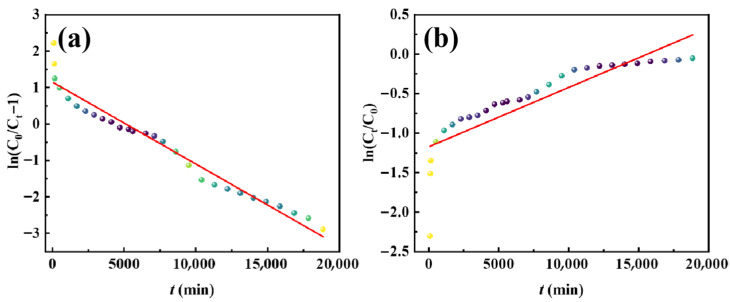
Thomas (**a**) and Adams-Bohart (**b**) model fitting curves for dynamic adsorption.

To evaluate the adsorption behavior, the Thomas (Equation (S9)) and Adams-Bohart (Equation (S10)) models [41,42,43] were employed to fit the experimental data (Figure 6). The Thomas model exhibited a strong correlation with the data (Table 6), with the calculated adsorption amount closely aligned with the theoretical value of 43.7 mg/g from static adsorption. This indicates that intraparticle diffusion is the primary mechanism governing the adsorption process. This is consistent with the material’s pore structure, which facilitates the diffusion of MB molecules into the adsorbent. In contrast, the Adams-Bohart model showed a lower correlation, indicating that its assumptions, such as mass transfer resistance and proportionality between adsorption rate and concentration, are less suited to this fixed-bed system.

Despite the promising results, potential errors could have influenced the experiment. These include variations in the flow rate of the peristaltic pump, which could affect the contact time between MB and the adsorbent, and uncertainties in the concentration analysis method. The dynamic adsorption capacity was calculated to be 42.0 mg/g, with an efficiency of 23.75%, lower than expected. This reduction in efficiency can be attributed to mass transfer resistance, which limits contact between MB molecules and active sites, as well as fixed-bed structural factors like non-uniform fluid distribution, flow rate issues, and packing density, which hinder optimal adsorption.

### 3.4. Adsorption Mechanism

**Figure 7:**
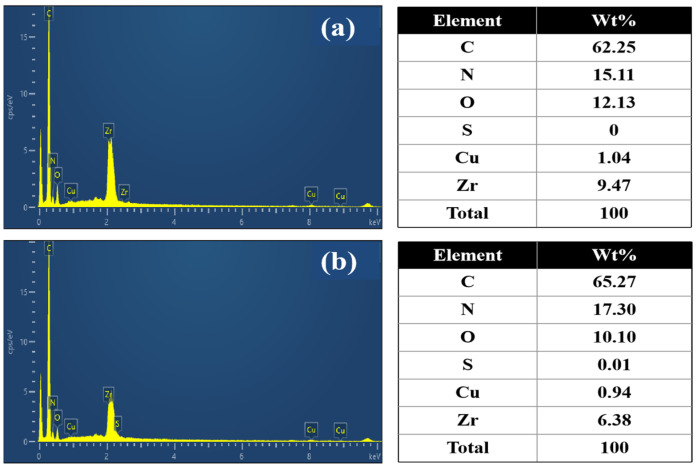
EDS analysis of Cu@Zr-MOFs-PAN before and after adsorption: (**a**) Cu@Zr-MOFs-PAN before adsorption, (**b**) Cu@Zr-MOFs-PAN after adsorption.

The EDS analysis (Figure 7) revealed that the Cu@Zr-MOFs-PAN was primarily composed of C, N, O, Cu, and Zr before adsorption. Following MB adsorption, the emergence of S element was observed, accompanied by an increase in C, N content, and a decrease in Cu, Zr content. These results clearly demonstrate the successful adsorption of MB onto Cu@Zr-MOFs-PAN. The infrared spectrum shows more serrated peaks in the range of 1586–1651 cm^−1^, corresponding to the C=C/C=N stretching vibrations of the benzene ring or nitrogen heterocycle in methylene blue. The enhancement of the peak at 1382 cm^−1^ further confirms the adsorption of methylene blue on Cu@Zr-MOFs-PAN. Additionally, the strengthening of the -C≡N stretching vibration peak at 2245 cm^−1^ may be attributed to the partial detachment of Cu@Zr-MOFs, exposing more -C≡N groups of PAN.

**Figure 8:**
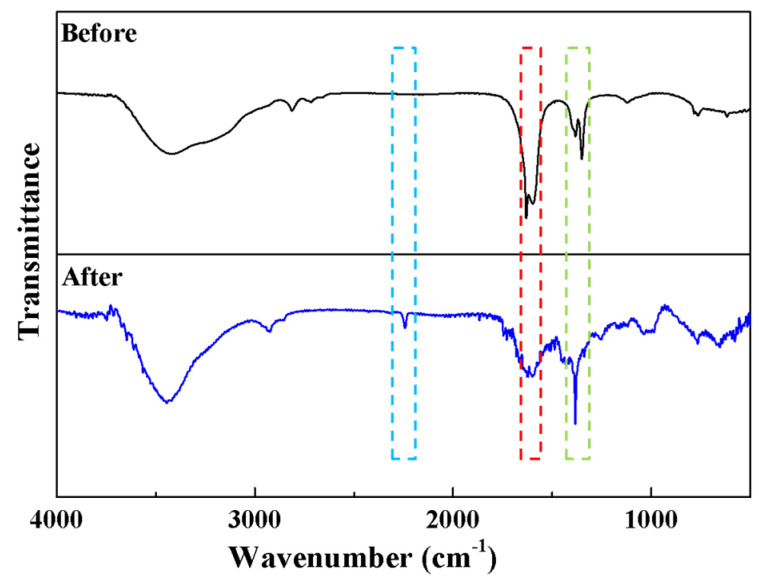
FT-IR analysis of Cu@Zr-MOFs-PAN before and after adsorption.

**Figure 9:**
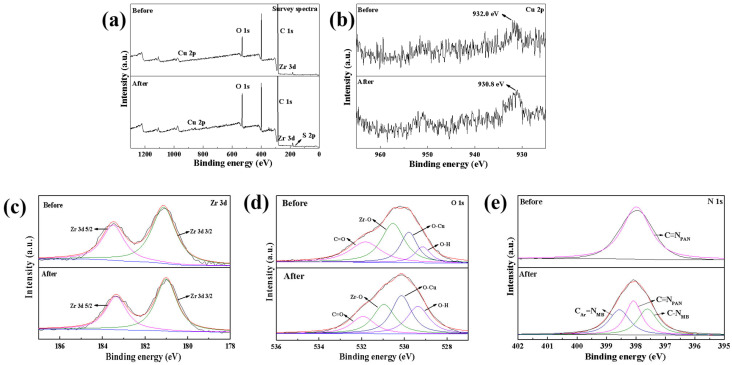
XPS survey spectra (**a**), Cu 2p spectra (**b**), Zr 3d spectra (**c**), O1s spectra (**d**) and N 1s spectra (**e**) of Cu@Zr-MOFs-PAN before and after the adsorption.

The XPS results before and after the adsorption of MB on Cu@Zr-MOFs-PAN are illustrated in Figure 8. As shown in Figure 9a, the detection of sulfur in the MB adsorbed on Cu@Zr-MOFs-PAN confirms the successful adsorption of the dye. Before adsorption, the binding energy of the 2p orbital of Cu^2+^ is measured at 932.0 eV, which shifts to 930.8 eV post-adsorption [2]. This decrease in binding energy suggests that Cu^2+^ participates in coordination interactions. The nitrogen atoms in the MB molecule, possessing lone-pair electrons, enable the formation of coordination bonds with Cu^2+^, thereby enhancing the adsorption efficiency.

Zr^4+^, typically forming Zr-MOFs in Cu@Zr-MOFs-PAN with TPA as a ligand, may also engage in coordination during the adsorption process due to local environmental changes, such as charge rearrangement or structural deformations induced by adsorption. Minor shifts in the binding energy of the Zr 3d orbitals (where the 3d_5_/_2_ peak shifts from 183.5 eV to 183.4 eV and the 3d_3_/_2_ peak from 181.1 eV to 181.0 eV post-adsorption) indicate its involvement in adsorption-related interactions [36], likely through electrostatic attraction or weak coordination with specific functional groups in the MB molecule.

The binding energy states of O 1s in Cu@Zr-MOFs-PAN are as follows: prior to adsorption, the binding energies are C=O at 531.8 eV, Zr-O at 530.5 eV, Cu-O at 529.8 eV, and O-H at 529.1 eV; after adsorption, they shift to C=O at 531.9 eV, Zr-O at 530.9 eV, Cu-O at 530.1 eV, and O-H at 529.3 eV. Notably, the increase in binding energies for both Cu-O and Zr-O suggests the formation or strengthening of these bonds during the adsorption process. The establishment of these bonds provides additional adsorption sites, enhancing the adsorption capacity for MB. The N1s spectrum (Figure 9e) of Cu@Zr-MOFs-PAN exhibits a single peak at 398.0 eV, corresponding to the C≡N_PAN_ group in PAN. After MB adsorption, the N 1s spectrum splits into three peaks assigned to C_Ar_=N (398.6 eV), C≡N_PAN_ (398.0 eV), and C-N_MB_ (397.6 eV). This indicates that the nitrogen in PAN (C≡N) retains its original binding energy (398.0 eV) and does not participate in the adsorption process of MB.

**Figure 10:**
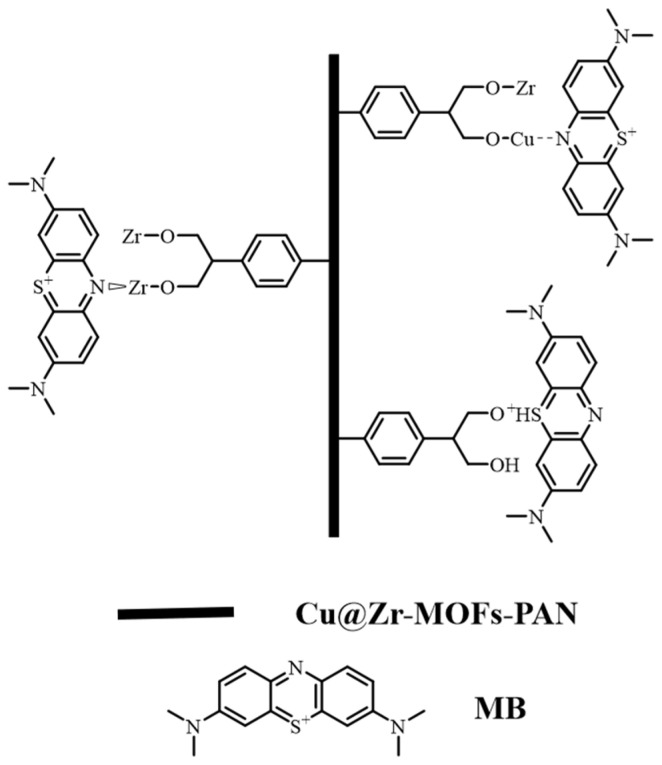
The possible adsorption mechanism of Cu@Zr-MOF-PAN for MB.

Based on the XPS analysis results, the adsorption mechanism of Cu@Zr-MOFs-PAN for MB primarily involves coordination and electrostatic interactions. Cu^2+^ plays a crucial role in coordination, enhancing the adsorption effect through its interaction with nitrogen atoms in MB. Concurrently, zirconium and oxygen contribute to the adsorption process mainly through electrostatic interactions. The positive charge on the surface of Zr oxide effectively attracts the negatively charged regions of the MB molecule, while the formation of Cu-O and Zr-O bonds further offers active sites for adsorption and enhances the overall adsorption capacity of the material (Figure 10).

## 4. Conclusions

In this study, Cu@Zr-MOF-PAN nanofiber composites were successfully prepared by doping copper ions into Zr-MOFs and embedding them in a PAN matrix. At pH = 7, the material exhibited the best adsorption performance for MB, with Cu@Zr-MOFs achieving an adsorption amount of 266.0 mg/g, substantially greater than that of undoped Zr-MOFs. Although the adsorption amount of Cu@Zr-MOF-PAN was 162.1 mg/g, it effectively utilized Cu@Zr-MOFs’ advantages and demonstrated a faster adsorption rate. Kinetic studies indicated that Zr-MOFs primarily utilized chemical adsorption, while Cu@Zr-MOFs and Cu@Zr-MOF-PAN exhibited a synergistic effect of physical and chemical adsorption, enhancing adsorption capacity. Isotherm model fitting revealed that all three materials followed the Langmuir model, and thermodynamic analysis revealed that the adsorption process was spontaneous and endothermic, with elevated temperatures promoting adsorption. In dynamic adsorption experiments, the Thomas model fitted well, with intraparticle diffusion being the dominant factor; however, due to mass transfer resistance, the actual dynamic adsorption capacity was lower than expected. XPS analysis confirmed that Cu^2+^ interacted with the nitrogen atoms in MB through coordination, while Zr^4+^ and oxygen participated in adsorption through electrostatic interactions. The formation of Cu-O and Zr-O bonds provided additional adsorption sites. Overall, this study provides an efficient adsorption material for dye wastewater treatment and innovatively demonstrates that copper ion doping significantly enhances the adsorption capacity of the material.

## Figures and Tables

**Table 1:** The N_2_ adsorption–desorption data of Zr-MOFs, Cu@Zr-MOFs, and Cu@Zr-MOFs-PAN.

Sample	SSA (m^2^/g)	PV (cc/g)	PS (nm)
Zr-MOFs	832.1	0.32	1.6
Cu@Zr-MOFs	435.0	0.29	2.6
Cu@Zr-MOFs-PAN	80.2	0.11	4.2

**Table 2:** The kinetic parameters for MB adsorption on Zr-MOFs, Cu@Zr-MOFs, and Cu@Zr-MOFs-PAN.

Materials	PFO Model			PSO Model		
	*q_e_*	*k*_1_	R^2^	*q_e_*	*k*_2_	R^2^
Zr-MOFs	89.2	0.012	0.876	96.8	1.8 × 10^−4^	0.998
Cu@Zr-MOFs	169.1	0.012	0.998	176.4	1.5 × 10^−4^	0.999
Cu@Zr-MOFs-PAN	121.9	0.022	0.995	124.2	5.8 × 10^−4^	0.999

**Table 3:** The data of the isothermal model for MB adsorption on Zr-MOFs, Cu@Zr-MOFs, and Cu@Zr-MOFs-PAN.

Materials	Langmuir Model			Freundlich Model		
	*q_m_*	*K_L_*	R^2^	*K_F_*	1/*n*	R^2^
Zr-MOFs	158.0	0.163	0.998	57.9	0.223	0.843
Cu@Zr-MOFs	266.0	0.351	0.999	145.5	0.137	0.983
Cu@Zr-MOFs-PAN	162.1	0.426	0.999	100.5	0.107	0.911

**Table 4:** Comparison of the maximum adsorption capacity (Q_m_) of Cu@Zr-MOFs-PAN with other adsorbents.

Materials	Adsorption Capacity	Reference
ZIF-8@strip 5/1 case	151.6 mg/g	[16]
UiO-66-PAMPS	120.3 mg/g	[31]
CSAC@AgNPs@TiO_2_NPs	184.0 mg/g	[5]
CMC/PAA/GO	138.4 mg/g	[3]
Cu@Zr-MOFs-PAN	162.1 mg/g	This work

**Table 5:** Thermodynamic parameters of MB adsorption on Zr-MOFs, Cu@Zr-MOFs, and Cu@Zr-MOFs-PAN.

Materials	Δ*G*^0^ (kJ/mol)			∆*H*^0^	∆*S*^0^ (J/mol K)
	288.15 K	298.15 K	308.15 K	(kJ/mol)	
Zr-MOFs	−3.4	−4.9	−6.3	38.2	144.6
Cu@Zr-MOFs	−6.1	−8.8	−11.5	70.2	265.1
Cu@Zr-MOFs-PAN	−4.5	−6.2	−7.8	42.2	162.3

**Table 6:** Related parameters of the two models.

Model	Parameter	Value
Thomas	*K_Th_*	1.9 × 10^−5^
	*q*_0_	43.7
	R^2^	0.978
Adams-Bohart	*k_AB_*	1.0 × 10^−5^
	*N*_0_	0.246
	R^2^	0.843

## Data Availability

The data presented in this study are available upon request from the corresponding author.

## Supplementary Materials

The following supporting information can be downloaded at: https://www.mdpi.com/article/10.3390/polym17172404/s1, Figure S1: N_2_ adsorption-desorption isotherms for Zr-MOFs, Cu@Zr-MOFs, and Cu@Zr-MOFs-PAN; Figure S2: Thermodynamic fitting curves for the adsorption of methylene blue by Zr-MOFs, Cu@Zr-MOFs, and Cu@Zr-MOFs-PAN.
