# Supplementary material for: Development of Cu@Zr-MOFs-PAN Nanofiber Composites for Efficient Methylene Blue Adsorption in Wastewater Treatment
Source: Polymers (Basel). 2025 Sep 3;17(17):2404. doi: 10.3390/polym17172404 (PMC12431038; doi:10.3390/polym17172404)
Supplement: Supplementary file 1 [file polymers-17-02404-s001.zip › polymers-3806619-supplementary.pdf]

polymers

MDPI

Development of Cu@Zr-MOFs-PAN Nanofiber Composites for Efficient Methylene Blue Adsorption in Wastewater Treatment

Zibin Li [1,*], Lizhen Zhang [1] and Guoyuan Yuan [2,*]

[1] School of Emergency Management, Chongqing Vocational Institute of Safety Technology, Chongqing 401331, China;
keyangongzuo521@163.com
[2] College of Chemistry and Chemical Engineering, Chongqing University of Science and Technology, Chongqing 401331, China
* Correspondence: lizibincq@126.com (Z.L.); guoyuan@cqust.edu.cn (G.Y.)

Section S1. Parameters of electrospinning
Section S2. Characterization equipment
Section S3. Dynamic adsorption
Section S4. Characterization analysis
Section S5. Adsorption kinetic
Section S6. Adsorption isothermal
Section S7. Adsorption thermodynamics
Section S8. Dynamic Adsorption

Section S1. Parameters of electrospinning

The electrospinning process was conducted under optimized conditions: an applied voltage of 16 kV and a precise flow rate of 0.0018 mL/h. To ensure complete solvent evaporation, the as-spun fibers were subjected to a controlled drying protocol at 60 °C for 12 hours. Subsequent immersion in deionized water facilitated the selective removal of PVP sacrificial components, yielding Cu@Zr-MOFs-PAN composite nanofibers with enhanced structural integrity.

Section S2. Characterization equipment

Comprehensive material characterization was performed to evaluate both structural and functional properties. X-ray diffraction (XRD) analysis (XRD-7000, Shimadzu, Kyoto, Japan) confirmed the crystalline structure, while Fourier transform infrared spectroscopy (FT-IR) (NICOLET iS10, Thermo Fisher Scientific, Waltham, MA, USA) identified surface functional groups. Brunauer-Emmett-Teller (BET) analysis (Autosorb iQ, Micromeritics, Norcross, GA, USA) quantified specific surface area, porosity distribution, and pore size characteristics. Scanning electron microscopy coupled with energy dispersive X-ray spectroscopy (SEM-EDS) (JSM-7800F, JEOL, Tokyo, Japan) revealed surface morphology and elemental composition. Finally, X-ray photoelectron spectroscopy (XPS) (Thermo Fisher Scientific, Waltham, MA, USA) tracked binding energy shifts of key elements before and after cobalt ion adsorption.

Section S3. Dynamic adsorption

$$q_{\mathrm{f}} = \frac{QA}{m \times 10^{3}}$$

(S1)

$$A = \int_{t=0}^{\mathrm{t=total}} C(t)\mathrm{dt}$$

(S2)

Here, the volumetric flow rate is denoted by Q (mL/min), while A represents the total adsorption capacity calculated from the area under the breakthrough curve (mg/L). The parameter m refers to the mass of the adsorbent (g), C(t) indicates the Co(II) concentration in the effluent at time t (mg/L), and t_{total} specifies the total operation time (min).

Section S4. Characterization analysis

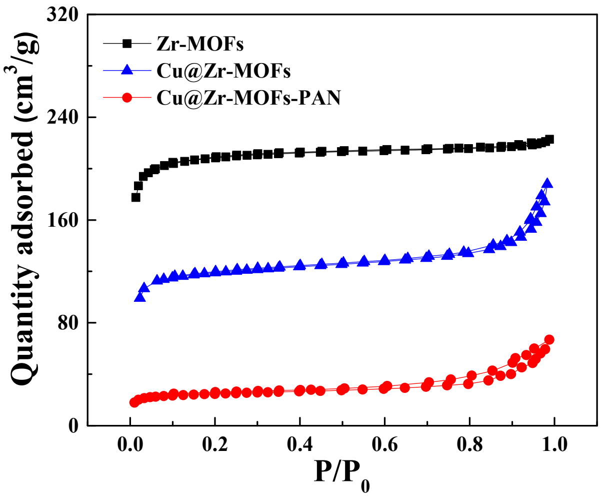

Figure S1. N_2 adsorption-desorption isotherms for Zr-MOFs, Cu@Zr-MOFs, and Cu@Zr-MOFs-PAN.

Section S5. Adsorption kinetic

The pseudo-first-order kinetic model (Equation (S3)), pseudo-second-order kinetic model (Equation (S4)) model as follows:

$$\ln\left(q_{\mathrm{e}} - q_{\mathrm{t}}\right) = \ln q_{\mathrm{e}} - k_{1}t \quad \text{(S3)}$$

$$\frac{t}{q_{\mathrm{t}}}=\frac{1}{k_2 q_{\mathrm{e}}^2}+\frac{t}{q_{\mathrm{e}}} \quad \text{(S4)}$$

In this context, k_1 (min^{-1}) and k_2 (g/mg·min) represent the rate constants for the pseudo-first-order and pseudo-second-order kinetic models, respectively. Additionally, q_t (mg/g) denotes the quantity of adsorption at a specific time *t*, while q_e (mg/g) signifies the equilibrium adsorption capacity.

Section S6. Adsorption isothermal

The Langmuir (Equation S5), Freundlich (Equation S6), isotherm models are as follows:

$$\frac{c_e}{q_e}=\frac{c_e}{q_m}+\frac{1}{q_m k_L} \quad \text{(S5)}$$

$$\ln q_e=\ln K_F+\frac{1}{n}\ln c_e \quad \text{(S6)}$$

In the equations, C_e (mg/L) represents the equilibrium concentration, q_e and q_m (mg/g) are the equilibrium adsorption amount and theoretical maximum adsorption capacity, K_L (L/mg) is the Langmuir equilibrium constant, K_F (mg g^{-1}(L mg^{-1})$^{1/n}$) and *n* are the Freundlich constants related to adsorption capacity and intensity.

Section S7. Adsorption thermodynamics

The thermodynamic model equations (Equation S7 and S8) are given below and the fitted curves are shown in Fig. S2:

$$\Delta G^0=\Delta H^0-T\Delta S^0 \quad \text{(S7)}$$

$$\ln K_d=\frac{\Delta S^0}{R}-\frac{\Delta H^0}{RT} \quad \text{(S8)}$$

In this context, *R* represents the universal gas constant, which has a value of 8.314 J/(mol·K). *T* denotes the temperature of the solution in K. K_d signifies the thermodynamic equilibrium constant. Furthermore, ΔH^0 corresponds to the change in enthalpy (measured in kJ/mol), ΔS^0 represents the change in entropy (expressed in J/(mol·K)), and ΔG^0 indicates the change in Gibbs free energy (given in kJ/mol).

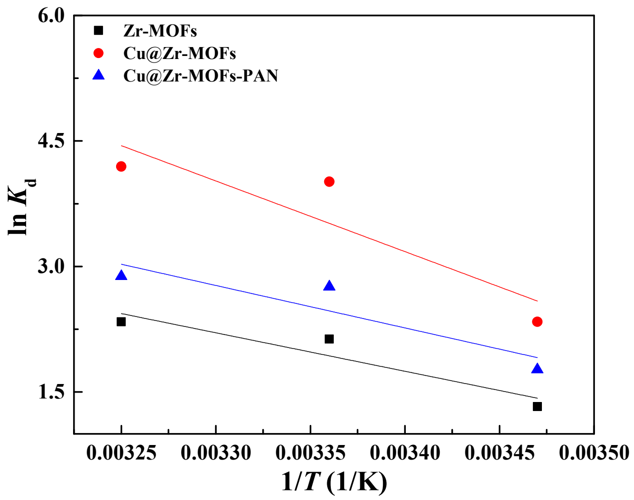

Figure S2. Thermodynamic fitting curves for the adsorption of methylene blue by Zr-MOFs, Cu@Zr-MOFs, and Cu@Zr-MOFs-PAN.

Section S8. Dynamic Adsorption

The fixed-bed adsorption model equations for Thomas (Equation S9) and Adams-Bohart (Equation S10) are given below:

$$\ln\left(\frac{C_t}{C_0}-1\right)=\frac{K_{Th}q_0 m}{Q}-K_{Th}C_0 t \tag{S9}$$

$$\ln\left(\frac{C_0}{C_t}\right)=K_{AB}C_0 t-K_{AB}N_0\left(\frac{Z}{U_0}\right) \tag{S10}$$

In this context, C_0 (mg/L) and C_t (mg/L) represent the initial and time-dependent ion concentrations, respectively. The Thomas model constant, K_{Th} (mL/(min·mg)), along with the adsorption capacity, q_0 (mg/g), the flow rate, Q (mL/min), and the total flow time, t (min), are integral components of the equation. K_{AB} (L/(mg·min)), the saturation concentration, N_0 (mg/L), and the column depth of the adsorption column, Z (cm), play crucial roles. The apparent velocity, U_0 (cm/min), is defined as the quotient of the volume flow rate.
